# Classification of Current Experimental Models of Epilepsy

**DOI:** 10.3390/brainsci14101024

**Published:** 2024-10-16

**Authors:** Carmen Rubio, Héctor Romo-Parra, Alejandro López-Landa, Moisés Rubio-Osornio

**Affiliations:** 1 Department of Neurophysiology, Instituto Nacional de Neurología y Neurocirugía, Mexico City 14269, Mexico; macaru4@yahoo.com.mx (C.R.); hector.romo.parra@gmail.com (H.R.-P.); alexlanda10@hotmail.com (A.L.-L.); 2 Psychology Department, Universidad Iberoamericana, Mexico City 01219, Mexico; 3 Department of Neurochemistry, Instituto Nacional de Neurología y Neurocirugía, Av. Insurgentes Sur 3877, Mexico City 14269, Mexico

**Keywords:** epilepsy models, chemical models, in vitro models, genetic models, physical models, focal epilepsy models, infectious models, developmental models

## Abstract

Introduction: This article provides an overview of several experimental models, including in vivo, genetics, chemical, knock-in, knock-out, electrical, in vitro, and optogenetics models, that have been employed to investigate epileptogenesis. The present review introduces a novel categorization of these models, taking into account the fact that the most recent classification that gained widespread acceptance was established by Fisher in 1989. A significant number of such models have become virtually outdated. Objective: This paper specifically examines the models that have contributed to the investigation of partial seizures, generalized seizures, and status epilepticus. Discussion: A description is provided of the primary features associated with the processes that produce and regulate the symptoms of various epileptogenesis models. Numerous experimental epilepsy models in animals have made substantial contributions to the investigation of particular brain regions that are capable of inducing seizures. Experimental models of epilepsy have also enabled the investigation of the therapeutic mechanisms of anti-epileptic medications. Typically, animals are selected for the development and study of experimental animal models of epilepsy based on the specific form of epilepsy being investigated. Conclusions: Currently, it is established that specific animal species can undergo epileptic seizures that resemble those described in humans. Nevertheless, it is crucial to acknowledge that a comprehensive assessment of all forms of human epilepsy has not been feasible. However, these experimental models, both those derived from channelopathies and others, have provided a limited comprehension of the fundamental mechanisms of this disease.

## 1. Introduction

Molecular causes and pathophysiological aspects of epilepsy are elucidated by experimental models of epileptogenesis [1,2,3,4]. These models are necessary for the development of new medicinal drugs and treatment processes [1,5,6,7]. In recent years, the Fisher (1989) epilepsy models have been less popular due to the emergence of more sophisticated and ethically sound models [8].

The penicillin model [9,10], devised by Walker and Johnson, entails the administration of penicillin at concentrations ranging from 1.7 to 3.4 nM to the cerebral cortex [11]. Even high doses of penicillin administered systemically can lead to brain seizures. Initially, Walker and Johnson (1945) demonstrated that this paradigm triggers epileptiform activity, which is distinguished by spikes in the electroencephalogram (EEG). It has been employed to induce myoclonic seizures and confirmed as a cat model of generalized seizures. This model primarily represents partial epilepsy [12]. Notwithstanding its extensive usage, the model has been employed less recently. Docksci and colleagues employed this approach in laboratory studies on mouse epileptogenesis in 2021, the last reference. A similar decline in popularity has been observed with the Curtis bicuculline model [13,14]. Administered systemically or topically to the brain of rats [15], it can induce localized epileptic seizures as a result of its actions as a blocker of GABA_A_ receptors [16,17,18]; despite its continued use, its frequency has decreased. The present work investigated the mechanisms of GABA_A_ receptor modulation in bicuculline-induced seizures by the bicuculline model, which by blocking calcium-dependent potassium channels, allows for excitability [19,20].

Specific studies employ the picrotoxin model [21,22,23]. Picrotoxin induced seizures by directly affecting GABA_A_R in the central nervous system (CNS) [24], therefore enhancing the excitability of neurons and promoting the release of epileptic discharges [25,26,27]. Advanced versions such as the pentylenetetrazole model have significantly decreased its usage [28,29]. Numerous recent investigations, such the one conducted in 2022, have utilized picrotoxin to examine inhibitory mechanisms and seizures [30]. An inherent limitation of this method is the widespread inhibition of GABA throughout the system. A well-established convulsive model for epileptogenic seizures is the strychnine model [31,32]. The toxic nature of strychnine prevents its use as a pharmaceutical agent [33]. The chemical in question was a pesticide and is well acknowledged for its toxicity and extensive experimental studies [34]. Glycine is a neurotransmitter with inhibitory properties that controls the excitability of neurons and maintains the balance of the CNS, therefore serving as a model for seizures [35,36,37]. Glycine may be inhibited by strychnine [38,39,40,41,42,43,44]. In recent times, this model has seen reduced usage, with specific toxicological investigations continuing to employ it until 2020. In comparison with more recent models, strychnine is less precise and has less medical relevance [45]. Aluminum and cobalt models implanted cortically have been discontinued due to ethical considerations and the progress made in developing less intrusive and more controlled techniques [46,47]. The aforementioned model has been sporadically referenced since the 1990s. Cobalt-induced seizure model enables epileptogenesis following the implantation of a cobalt wire in the cerebral cortex, forming a persistent epileptic focus [48,49,50,51]. By the 2000s, the literature no longer recorded the use of cobalt. Existing articles cite previous research rather than direct use of the model, as it has been replaced by techniques that offer greater control and replicability without the need for metal implants, thus minimizing associated complications [52].

While acute electrical stimulation is used in some research, chronic techniques like kindling [53,54], which involve applying sub-threshold impulses to limbic areas to prolong and complicate seizures, are becoming less prevalent because of their excessive technical complexity [55,56]. This approach has produced remarkable outcomes and is still employed by certain researchers [57,58,59,60,61,62]. In their 2024 publication, Jiang et al. present the “Electric Kindling Model for Studying Drug-Resistant Epilepsy”. The kindling model was employed to investigate drug-resistant epilepsy, uncovering novel mechanisms and therapeutic interventions [63,64,65]. While older models remain valuable for investigating certain aspects of epilepsy, the field of epilepsy research is increasingly shifting towards more contemporary and targeted methodologies for this reason. This model is partially out of use. Genetic, optogenetic, and selective toxin models are highly specific and suitable for studying particular variants of epilepsy, allowing for the development of more effective therapies while reducing associated risks and ethical concerns [66,67]. This has led to the abandonment of penicillin, picrotoxin, strychnine, aluminum, and cobalt models. Recent advancements in these models enable a more personalized methodology and often provide more accurate simulations of human epilepsy pathophysiology. In this review, we classify the most representative and significant epilepsy models and acute seizure models induced by chemoconvulsants, those that have paved the way for new research avenues and contributed to advancing therapies for the various types of epilepsy present in humans.

## 2. In Vivo Models

### 2.1. Genetics Models

#### 2.1.1. Gerbils

First devised by Smith in 1924 [68], the audiogenic seizure paradigm causes seizures in gerbils by subjecting them to high-intensity sound stimuli [69]. Gerbils are the predominant species employed in this paradigm because of their reliable reaction to auditory stimulation accompanied by seizure activity [70]. Nevertheless, individual strains of gerbils exhibit varying degrees of vulnerability to audiogenic seizures, which has supported their use in epilepsy research [71]. Auditory stimuli within the range of 102 to 131 dB elicit a convulsive reaction marked by tonic flexion followed by the loss of tonic extension [72,73,74,75]. Subsequently, a postictal condition characterized by exploratory behavior takes place [76].

#### 2.1.2. The Photosensitive Baboon Model (*Papio papio*)

The Photosensitive Baboon Model is defined by a genetic predisposition to seizures triggered by light stimuli [77,78]. In this model, tonic–clonic seizures are induced through exposure to stroboscopic stimuli, particularly at a frequency of 25 Hz [79]. During stimulation, the baboons may experience generalized seizures lasting between 20 and 30 s [80,81]. These seizures manifest as generalized myoclonic discharges, primarily affecting the occipital and frontocentral regions of both cerebral hemispheres, depending on the individual’s sensitivity [82,83,84,85]. The early and intense spike and polyspike patterns observed in the frontocentral areas of both hemispheres are associated with the convulsive discharges in these baboons. The electroencephalographic patterns in this model are characteristic of photosensitive epilepsy [86].

#### 2.1.3. Scn1a Gene Models

For the *Scn1a* gene knock-in and knock-out models, both approaches are used to study epilepsy, particularly Dravet syndrome [87,88,89]. In the knock-in model, a mutated version of the *Scn1a* gene, which encodes a sodium channel (NaV1.1) [90], is introduced. This mutation reduces the function of inhibitory neurons (interneurons), leading to hyperexcitability and a lowered seizure threshold [91]. In the knock-out model, the *Scn1a* gene is completely inactivated, resulting in severe epilepsy due to the loss of sodium channel function, which is crucial for maintaining the balance between excitatory and inhibitory signals in the brain [92]. Both models demonstrate how disruptions in sodium channel function contribute to epileptic activity, providing insight into the pathophysiology of genetic epilepsy [93].

These models are essential for assessing the safety and effectiveness of new pharmacological compounds that can modify sodium channel activity, as well as for studying sodium channel dysfunction [94]. This is particularly significant for the development of more effective anti-epileptic drugs with fewer side effects. Recent research using these models has demonstrated how changes in NaV1.1 expression can influence the function of other ion channels and receptors in the brain [7]. These findings also highlight the interaction between sodium channel dysfunction and other neural systems [91,95]. Comprehensive studies have shown that pharmacological restoration of NaV1.1 function can correct certain electrophysiological and behavioral abnormalities in mouse and zebrafish models, paving the way for targeted therapies in humans [27].

#### 2.1.4. Scn2a Gene Model

The *Scn2a* gene encodes the alpha subunit of the voltage-gated sodium channel type II (NaV1.2) [96], which is crucial for generating and propagating action potentials in neurons. Mutations in this gene are associated with various forms of epilepsy, including early-onset epilepsy and other neurological syndromes [97,98,99,100]. Animal models and clinical studies of *Scn2a* have been key in understanding how sodium channel dysfunctions contribute to epilepsy [99,101]. Animal models, particularly in mice, have been essential for studying *Scn2a* mutations and their effects on neuronal excitability and seizure behavior. Knock-in mice with gain-of-function mutations in *Scn2a* show increased neuronal firing and heightened susceptibility to induced seizures [102]. These models are useful for studying early-onset epilepsies characterized by hyperactive sodium channels [103]. Knock-out mice or those with loss-of-function mutations in *Scn2a* display reduced neuronal firing and often exhibit abnormal brain development, resulting in cognitive deficits and milder seizures. These models are important for investigating the spectrum of neurological disorders related to autism and epilepsy [104]. The studies on mouse models with *Scn2a* mutations have revealed the alterations in sodium channel activation threshold. *Scn2a* mutations affect the activation and inactivation thresholds of NaV1.2 sodium channels, directly influencing the ability of neurons to generate action potentials [105,106]. Dysfunction in NaV1.2 channels can lead to abnormal signal propagation in neural networks, contributing to neuronal desynchronization and seizure onset. NaV1.2 channels are predominantly expressed in excitatory pyramidal neurons, particularly in dendrites and axons. Mutations in *Scm2a* primarily affect these neurons, leading to excessive excitability in cortical neural networks [107,108]. GABAergic interneurons are not directly affected, but dysfunction in excitatory neurons disrupts the balance between excitation and inhibition, promoting epileptic activity.

Mutations in *Scn2a* are linked to several epileptic syndromes and neurological disorders, including, early infantile epileptic encephalopathy (EIEE11), a severe epileptic disorder that begins in the first few months of life, associated with frequent seizures, developmental delays, and cognitive dysfunction [109,110]. Gain-of-function mutations in *Scn2a* are common in this condition. Benign familial infantile seizures. A milder form of epilepsy is linked to *Scn2a* mutations, typically presenting with focal seizures in infancy and a better prognosis. Autism spectrum disorder and intellectual disability: Some loss-of-function mutations in *Scn2a* are associated with autism and developmental delays without severe epilepsy. *Scn2a* epilepsy models have been crucial for developing targeted therapies that modulate sodium channel activity [111,112]. Some therapeutic approaches include sodium channel blockers, drugs such as phenytoin and lamotrigine, which block sodium channel activity, and can be effective in controlling seizures in patients with gain-of-function *Scn2a* mutations.

#### 2.1.5. Scn8a Gene Model

The *Scn8a* D/+ or *N1768D* mouse model is based on a mutation in the *Scn8a* gene, which encodes the voltage-gated sodium channel type 8 (Nav1.6) [113]. This channel is crucial for the generation and propagation of action potentials in neurons, particularly in brain regions associated with neuronal excitability [114]. Mice with the *N1768D* mutation are valuable for studying epilepsy, as this alteration affects Nav1.6 function, leading to increased neuronal excitability and the occurrence of spontaneous seizures [115]. The mutation in *Scn8a* results in a gain-of-function effect in the Nav1.6 sodium channel, causing an increase in sodium influx into neurons [116]. This, in turn, lowers the excitability threshold, making it easier for neurons to generate and propagate action potentials. Nav1.6 channels are essential in axons and at the nodes of Ranvier, where they facilitate the rapid transmission of electrical signals along neurons [117]. As a consequence of the enhanced activity of Nav1.6 channels, neurons become hyperexcitable and prone to repetitive, prolonged discharges [113]. This heightened excitability increases the likelihood of epileptic seizures, as synaptic discharges become unregulated. *Scn8a* D/+ mice exhibit spontaneous seizures that vary in intensity and frequency, including focal seizures, generalized tonic–clonic seizures, and non-motor seizures [118]. These spontaneous seizures mimic certain human genetic epilepsies, such as *Scn8a*-related epilepsy, which is associated with Nav1.6 dysfunction [119].

Prolonged neuronal hyperactivity can also lead to neurodegeneration in some brain regions, as excessive excitation triggers excitotoxicity, a process in which overexcited neurons undergo cell death. Additionally, some studies have reported cognitive deficits in Scn8a mutant mice, possibly due to alterations in synaptic plasticity, such as impaired long-term potentiation (LTP), a critical mechanism for learning and memory [120]. This mouse model is widely used to investigate the pathophysiology of genetic epilepsies and the role of ion channel mutations in driving epileptic activity [121]. It serves as a valuable tool for exploring potential treatments targeting sodium channels to manage seizure disorders more effectively.

#### 2.1.6. Kcna1 KO Model

The Kcna1 KO (knock-out) model involves the deletion of the *Kcna1* gene [122], which encodes the alpha subunit of the voltage-gated potassium channel Kv1.1. These channels play a crucial role in regulating neuronal excitability by mediating potassium efflux, helping neurons repolarize and stabilize their membrane potential after an action potential [123]. In the absence of the *Kcna1* gene in knock-out mice, the ability of neurons to regulate their excitability is significantly impaired, leading to increased neuronal excitability and a predisposition to frequent seizures, hyperexcitability, and other neurological phenotypes [124]. This model is widely used to study epilepsy and disorders linked to dysregulated neuronal excitability. This hyperexcitability particularly affects regions such as the hippocampus, thalamus, and cerebral cortex, which are important for seizure propagation [125].

Kcna1 KO mice experience spontaneous seizures ranging from focal to generalized tonic–clonic seizures [126]. The frequency of seizures tends to increase as the mice age, often resulting in premature death due to uncontrolled seizures. This makes the Kcna1 KO model useful for studying severe epilepsy forms, particularly those associated with genetic mutations affecting ion channels. In addition to epilepsy, this knock-out model is also pivotal in research on Sudden Unexpected Death in Epilepsy (SUDEP) [122]. Mice lacking *Kcna1* are prone to sudden death, which is linked to cardiac and respiratory failure following seizures. This characteristic makes the model valuable for understanding the mechanisms underlying SUDEP and for exploring potential therapeutic interventions aimed at preventing sudden death in epilepsy patients.

#### 2.1.7. Cacna1a Gene Model in Mouse (Tottering) and Zebrafish

The Tottering Mouse Model is based on a mutation in the *Cacna1a* gene, which encodes the Alpha 1A subunit of P/Q-type calcium channels [127]. These channels are essential for regulating calcium entry into cells, particularly at neuronal synapses, where they influence neurotransmitter release [128]. Mutations in *Cacna1a*, such as the one found in tottering mice, lead to dysfunction in these channels and are associated with various neurological disorders, including ataxia, epilepsy, and migraines. In this model, the mutation in P/Q-type calcium channels disrupts calcium currents at synaptic terminals [70]. This impairment affects the proper release of neurotransmitters, especially in inhibitory interneurons that utilize GABA [129]. As a result, there is an imbalance between excitatory and inhibitory signaling in the nervous system. One of the most noticeable features of tottering mice is ataxia, characterized by motor incoordination and tremor-like movements [130]. This is due to cerebellar dysfunction, where the lack of adequate neurotransmitter release in cerebellar synapses impairs motor control and postural stability. In addition to ataxia, tottering mice develop spontaneous seizures, which may be linked to a decrease in GABA release [131]. The reduced inhibitory signaling tips the balance toward neuronal hyperexcitability, making the brain more prone to seizures. These seizures vary in severity and can range from tonic–clonic to absence seizures. Furthermore, *Cacna1a* mutations in humans are linked to familial hemiplegic migraine type 1 (FHM1), and tottering mice exhibit related phenotypes, such as stress hypersensitivity and impaired regulation of cerebral blood flow [132].

The tottering mouse model is used to investigate several neurological disorders. It is a key tool in epilepsy research, allowing scientists to study the mechanisms underlying seizures and how calcium channel dysfunction contributes to neuronal hyperexcitability. It is also an important model for hereditary ataxias, helping to understand cerebellar abnormalities that lead to motor dysfunction [131,133,134,135]. Additionally, the model is used in pharmacological studies to test drugs that may improve motor function, reduce seizure frequency, or alleviate migraine symptoms. Overall, tottering mice provide critical insights into how mutations in the *Cacna1a* gene and P/Q-type calcium channels contribute to complex neurological disorders such as ataxia, epilepsy, migraines, and other syndromes involving dysregulated neuronal signaling [131].

On the other hand, zebrafish (*Danio rerio*) are promising organisms that have recently emerged for studying various brain disorders, such as epilepsy [136], due to their phylogenetic and physiological similarities with mammals and humans. Furthermore, the wide range of molecular, genetic, and electrophysiological manipulation techniques has enabled these organisms to surpass traditional epilepsy models [137,138]. 

In 2020, the *cacna1aa* phenotype was characterized for the first time in larval zebrafish [139]. This same group found a duplication of the *cacna1a* gene, sharing 72.01% homology in *cacna1aa* and 71.28% in *cacna1ab* with the human *CACNA1A* gene. Their electroencephalogram (EEG) analysis demonstrated that 92% of the zebrafish exhibited epileptiform-like events, which were compatible with abrupt spike-wave complexes, polyspike wave discharges, and high-voltage spikes. Based on the pattern of behavioral changes, they predicted that the *cacna1aa*-related epileptic phenotype may involve absence seizures [139]. Their results could open useful avenues for elucidating epileptogenic mechanisms mediated by this gene in absence seizures.

#### 2.1.8. The Stargazer Mouse Model

The Stargazer Mouse Model is based on a mutation in the *Cacng2* gene, which encodes the stargazin protein, an auxiliary subunit of AMPA-type calcium channels and voltage-gated calcium channels [140]. Stargazin plays a critical role in the proper localization and function of AMPA receptors at the synapse, which are involved in fast excitatory synaptic transmission in the central nervous system. The mutation in *Cacng2* interferes with the expression and function of these receptors, affecting excitatory signaling in the brain [141]. Stargazer mice are characterized by spontaneous seizures, primarily of the absence type, where brief lapses in consciousness and motor activity occur. This makes the model valuable for studying absence epilepsy [142]. The reduction in AMPA receptor activity in brain regions responsible for excitatory synaptic transmission, such as the cortex and thalamus, contributes to the development of seizures [143]. Additionally, Stargazer mice display ataxia, or motor incoordination, due to impaired synaptic transmission in the cerebellum, a region critical for balance and motor control. The mutation in *Cacng2* affects AMPA receptor function in this area, leading to movement difficulties [144]. Another notable phenotype in Stargazer mice is deafness, as the mutation disrupts synaptic connections in the auditory system, hindering the brain’s ability to process sound. This mouse model has been extensively used to explore the cellular and molecular mechanisms underlying epilepsy, ataxia, and sensory disorders [145]. It provides valuable insights into how alterations in calcium channels and glutamate receptors contribute to brain dysfunctions and neurological diseases [146].

#### 2.1.9. The Ducky (Ddu) Mouse Model

The Ducky Mouse Model involves a mutation in the *Cacna2d2* gene [147], which encodes the α2δ2 subunit of voltage-gated calcium channels [148]. This subunit is critical for the proper functioning of calcium channels, as it plays a key role in stabilizing the channels and regulating calcium flow into neurons. The mutation in Cacna2d2 in Ducky mice leads to disruptions in calcium signaling, which is essential for neurotransmitter release, synaptic plasticity, and overall neuronal excitability [149]. Ducky mice display several neurological phenotypes, most notably epilepsy and ataxia. The mutation impairs calcium channel function, leading to seizures due to abnormal neuronal firing and excitability [149]. These seizures are primarily of the absence type, characterized by brief lapses in consciousness. Additionally, Ducky mice exhibit ataxia, or lack of coordination, due to the involvement of calcium channels in cerebellar function. The cerebellum, which plays a crucial role in motor control and coordination, is particularly affected by the dysfunctional calcium channels in this model [150]. The *Cacna2d2* mutation disrupts the trafficking and functioning of calcium channels at synapses, which impairs neurotransmitter release, leading to the abnormal neuronal signaling seen in both the epileptic seizures and the motor deficits characteristic of Ducky mice [151,152]. This model is valuable for studying the role of calcium channels in epilepsy and ataxia and provides insights into how calcium channel dysfunction can lead to neurological disorders.

#### 2.1.10. Gabra1 Gene Model

The Gabra1 Gene Model involves a mutation in the *Gabra1* gene [153], which encodes the α1 subunit of the GABA_A_ receptor. This receptor is a ligand-gated ion channel that plays a critical role in inhibitory neurotransmission within the central nervous system. It operates by allowing chloride ions to enter neurons, leading to hyperpolarization and reduced neuronal excitability [154]. In the *Gabra1* knock-out or mutant mouse models, the absence or dysfunction of this receptor subunit results in several significant neurological abnormalities [155]. The primary phenotype observed in these mice is epilepsy. The lack of functional GABA_A_ receptors leads to reduced inhibitory control over neuronal activity, causing increased excitability and spontaneous seizures [156]. These seizures can manifest in various forms, including both focal and generalized types. This model is instrumental in studying how deficits in inhibitory signaling contribute to the onset and progression of seizures. Additionally, the absence of the α1 subunit disrupts the normal functioning of GABA_A_ receptors, resulting in decreased inhibitory neurotransmission and a state of neuronal hyperexcitability. This heightened excitability is a central factor in the development of seizures and epilepsy observed in this model. Gabra1 mutant mice also often exhibit behavioral and cognitive impairments due to the extensive disruption of inhibitory signaling in the brain [157]. These impairments can include anxiety-like behaviors, altered locomotion, and compromised learning and memory abilities. Furthermore, the absence of the α1 subunit can impact the development of inhibitory circuits in the brain, potentially leading to long-term deficits in synaptic function and connectivity. This has implications for understanding the developmental aspects of epilepsy and related disorders. Overall, the *Gabra1* mutant mouse model is a valuable tool for investigating the role of GABA_A_ receptors in epilepsy and understanding how impaired inhibitory signaling contributes to seizure disorders. It also serves as a model for exploring potential therapeutic interventions aimed at restoring GABA_A_ receptor function or compensating for its loss [158].

In the case of zebrafish, the homologous damaged gene is the GABRA1A gene. According to Kralic et al. (2005) [159], knock-out mice only exhibited tremors and never displayed an epileptic phenotype. Consequently, in 2018, Samarut et al. [160] generated a new line of *gabra1a* mutant zebrafish, which showed 83% homology with the human protein. Upon light exposure, the zebrafish exhibited fully penetrant tonic–clonic seizures. An analysis of the structures containing Gad1/2 in the mutant brains revealed a reduction in Gad1/2-labeled neurofilaments, suggesting decreased presynaptic GABAergic signaling. This was accompanied by a reduction in the expression of gephyrin-positive clusters in the mutant brains, demonstrating that GABA is essential for proper communication within this inhibitory synaptic network.

#### 2.1.11. The Grin2a

The Grin2a receptor, specifically its NMDA (N-methyl-D-aspartate) subtype, plays a crucial role in excitatory neurotransmission in the central nervous system [161]. Mutations in the *Grin2a* gene, which encodes the NMDA receptor’s NR2A subunit, have been associated with a range of neurological disorders, including focal epilepsy and epileptic encephalopathy. In models with mutations in the *Grin2a* gene, the NMDA receptors often exhibit altered functionality [162]. These receptors are integral for synaptic plasticity, learning, and memory processes, and their dysfunction can lead to significant neurological and cognitive disturbances. One of the primary manifestations in these models is focal epilepsy, characterized by seizures that originate in a specific area of the brain before potentially spreading to other regions [163]. The impaired NMDA receptor function results in disrupted excitatory–inhibitory balance, which contributes to increased neuronal excitability and the development of seizures [164].

Moreover, mutations in the *Grin2a* gene are also linked to epileptic encephalopathy, a severe form of epilepsy that presents with early onset, frequent seizures, and significant developmental and cognitive impairments. This condition is marked by a progressive decline in cognitive and motor functions, which can severely impact the quality of life. Epileptic activity in this model and the other expressed gene models is often resistant to standard anti-epileptic treatments, highlighting the complex interaction between NMDA receptor dysfunction and epilepsy [165]. Research using *Grin2a* mutant models is instrumental in understanding the pathophysiology of focal epilepsy and epileptic encephalopathy. These models provide insights into how alterations in NMDA receptor function contribute to abnormal neuronal activity and seizure generation [166]. They also offer a platform for evaluating potential therapeutic strategies aimed at modulating NMDA receptor activity or compensating for its dysfunction. Overall, the *Grin2a* receptor mutant models are valuable tools for unraveling the mechanisms underlying these severe epilepsy forms and developing targeted interventions [167].

#### 2.1.12. The PTEN Gene Is Associated with Epilepsy Related to Tuberous Sclerosis Complex

The *PTEN* gene is crucial for regulating cell growth and maintaining cellular homeostasis through its role as a tumor suppressor [168]. Mutations or deletions in the PTEN gene have been linked to a variety of neurological and developmental disorders, including epilepsy associated with tuberous sclerosis complex (TSC) [169]. Tuberous sclerosis complex is a genetic disorder characterized by the growth of non-cancerous tumors in multiple organs, including the brain. This condition is often associated with epilepsy, particularly in the context of *PTEN* gene mutations. The *PTEN* gene mutations lead to a disruption in its normal tumor-suppressive function, which results in uncontrolled cell growth and the formation of cortical tubers—abnormal masses of brain tissue [169].

In animal models with *PTEN* gene mutations, these cortical tubers manifest as focal malformations that contribute to the development of focal epilepsy [170]. The abnormal brain tissue disrupts normal neuronal circuits and increases neuronal excitability, leading to the generation of seizures that typically start in specific brain regions. These focal seizures can be complex, often evolving into secondary generalized seizures [171]. The *PTEN* mutation models also reveal insights into the broader impact of TSC on brain function. Beyond seizures, individuals with tuberous sclerosis often experience a range of cognitive and developmental impairments due to the widespread presence of cortical tubers [172]. These models help researchers understand how the abnormal brain tissue affects cognitive and motor functions, and they highlight the challenges in managing epilepsy that is resistant to standard treatments. Overall, the *PTEN* gene mutation models are essential for studying the mechanisms underlying epilepsy associated with the tuberous sclerosis complex [173]. They provide valuable information about how disrupted cellular regulation and brain malformations contribute to seizure generation and progression. Additionally, these models are crucial for testing potential therapeutic approaches aimed at managing seizures and mitigating the cognitive impacts of the disorder.

#### 2.1.13. Ch2 Gene Model

Mutations in the Ch2 gene (which encodes the chromatin helicase DNA-binding protein domain 2 in humans) are associated with a range of epileptic disorders, including Lennox–Gastaut syndrome, myoclonic–atonic epilepsy, and Dravet syndrome, and are often linked to intellectual disability and developmental delays [174,175,176,177]. Although these mutations are not directly related to ionic conductance or synaptic transmission, they do affect the expression, recombination, and repair of DNA, as well as cell cycle regulation and differentiation. In rodents, the Chd2 gene demonstrated developmental abnormalities, but no epileptiform phenotypes were confirmed as seen in humans. The Chd2 gene in zebrafish has a 70% homology with the human CHD2 protein. In 2013, Suls et al. [176] showed that mutations in the Chd2 gene of zebrafish led to behavioral abnormalities at 4 days post-fertilization (dpf) that suggested epileptic seizures, such as burst activity, pectoral fin and jaw contractions, and whole-body tremors. In 2015, Galizia et al. [175] quantified the duration and frequency of discharges during a period of darkness followed by light exposure, demonstrating a higher frequency and longer duration of discharges compared to controls.

### 2.2. Chemical Models

#### 2.2.1. Kainic Acid Model

Kainic acid (KA) is a potent excitatory amino acid derived from kainate, a naturally occurring compound. It is commonly used in experimental models of rodents of different ages and in adult zebrafish to induce temporal lobe epilepsy and study related neurological conditions [178]. KA acts as an agonist at the kainate subtype of glutamate receptors, which are involved in excitatory neurotransmission in the CNS [179]. When administered in research settings, KA induces seizures by overstimulating glutamate receptors, leading to excessive neuronal excitation and eventually causing seizures [180]. This model is particularly valuable for studying temporal lobe epilepsy, a type of epilepsy characterized by recurrent seizures originating from the temporal lobe of the brain [181].

The application of KA in experimental models also results in selective neuronal death, which is a key feature of this model. The intense excitatory signaling triggered by KA leads to excitotoxicity, where excessive glutamate release causes neuronal injury and death [182]. This selective neuronal loss primarily affects the hippocampus and surrounding temporal lobe structures, which are critical regions involved in memory and emotional regulation [183]. The KA model is instrumental in understanding the mechanisms of temporal lobe epilepsy and the processes underlying selective neuronal damage. It helps researchers explore how excessive excitatory neurotransmission contributes to seizure generation and neuronal loss [184]. Additionally, this model provides insights into potential therapeutic strategies aimed at mitigating excitotoxic damage and improving treatment outcomes for temporal lobe epilepsy and related disorders.

#### 2.2.2. Pentylenetetrazole

Pentylenetetrazole (PTZ) is a chemical compound known for its role as an antagonist of the GABA_A_ receptor [185], a key player in inhibitory neurotransmission within the central nervous system. In experimental models of rodents and zebrafish, PTZ is used to induce generalized seizures, which are widespread seizures affecting the entire brain [186]. By blocking the GABA_A_ receptors, PTZ disrupts the normal inhibitory signaling in the brain. GABA_A_ receptors are crucial for maintaining the balance between excitatory and inhibitory neurotransmission, and their inhibition leads to a reduction in inhibitory control. This disruption results in increased neuronal excitability and susceptibility to seizures [187]. The PTZ-induced seizure model is valuable for studying generalized seizures and understanding the underlying mechanisms of seizure propagation. It provides insights into how impaired inhibitory signaling contributes to the development and spread of seizures throughout the brain [188]. Additionally, this model is used to evaluate the efficacy of potential anti-epileptic drugs and therapeutic interventions aimed at restoring balance between excitatory and inhibitory signaling. Overall, the PTZ model is a widely used tool in epilepsy research, helping scientists explore the effects of GABA_A_ receptor antagonism on seizure activity and assess strategies for managing generalized seizures [189].

PTZ is considered to induce a similar reaction in both rodents and zebrafish, serving as a model for generalized seizures, particularly absence-like and generalized tonic–clonic seizures. However, PTZ enabled zebrafish to be used as an epilepsy model for the first time in 2005 by Baraban et al. [190]. In this study, they approximated the characteristics of the PTZ model in zebrafish larvae, including behavioral and extracellular electrical activity [138]. This work laid the groundwork for the future development of zebrafish epilepsy models.

### 2.3. Electrical Models

#### Kindling

Kindling is an experimental model of epilepsy that involves the gradual and progressive development of seizures through repeated, sub-threshold electrical stimulation of the brain [191]. This model provides valuable insights into the mechanisms underlying epilepsy and the process of seizure sensitization. In the kindling model, low-intensity electrical stimuli are applied to specific brain regions, typically the amygdala or hippocampus, at regular intervals [192]. Each stimulation event is not sufficient to induce a full-blown seizure on its own but contributes to a cumulative effect over time. As the number of simulations increases, the brain becomes increasingly responsive to the stimuli, leading to the gradual emergence of more severe and prolonged seizures. This progressive increase in seizure severity and frequency reflects the phenomenon of “kindling,” where repeated exposure to sub-threshold stimuli leads to the enhancement and consolidation of seizure activity [193]. The model is used to study various aspects of epilepsy, including the processes involved in seizure initiation, propagation, and the development of chronic epilepsy [54]. It also helps in understanding how neuronal networks become sensitized to epileptogenic stimuli. The kindling model is particularly valuable for investigating the mechanisms of epileptogenesis, the process by which a normal brain develops epilepsy—and for evaluating the efficacy of anti-epileptic drugs and other therapeutic interventions. By simulating the gradual development of epilepsy, kindling provides insights into the long-term changes that occur in the brain and helps researchers identify potential targets for preventing or treating epilepsy [54].

### 2.4. Structural Models

#### Hippocampal Sclerosis

Hippocampal sclerosis is a condition characterized by the degeneration and loss of neurons specifically within the hippocampus, a crucial brain region involved in memory formation and spatial navigation [194,195]. This model is frequently used to study temporal lobe epilepsy, a type of epilepsy that originates in the temporal lobe of the brain. In the hippocampal sclerosis model, injury to the hippocampus is typically induced through various means, such as seizures, inflammation, or genetic mutations [196]. The resulting neuronal damage and loss lead to a significant disruption in the hippocampal structure and function [197]. This damage is often associated with the development of temporal lobe epilepsy [198]. The primary feature of this model is the selective and progressive loss of hippocampal neurons, which contributes to the generation of seizures. The degeneration of neuronal circuits within the hippocampus leads to an imbalance between excitatory and inhibitory signaling, resulting in increased neuronal excitability and the onset of seizures. These seizures can be focal, originating from the damaged hippocampal region, and may spread to other parts of the brain [199]. Hippocampal sclerosis is a valuable model for understanding the pathophysiology of temporal lobe epilepsy and for investigating the mechanisms of seizure generation and propagation [200]. It provides insights into how structural changes in the hippocampus contribute to epilepsy and helps researchers explore potential therapeutic strategies aimed at protecting hippocampal neurons or restoring normal brain function. Additionally, this model is instrumental in studying the cognitive and memory impairments that often accompany temporal lobe epilepsy, offering a comprehensive view of the impact of hippocampal damage on overall brain function.

## 3. In Vitro Models

### 3.1. Hippocampal Slices

Hippocampal slices involve isolating and studying thin sections of hippocampal brain tissue to investigate epileptic activity [201]. This model is particularly useful for examining the intrinsic properties of the hippocampus and understanding how epileptic activity arises and propagates within this critical brain region. In this model, the hippocampus is carefully dissected from the brain and sliced into thin sections, which are then maintained in a controlled environment that mimics physiological conditions [202]. These hippocampal slices are placed in an artificial cerebrospinal fluid solution, which supports the viability of the tissue and allows for detailed electrophysiological recordings and other experimental manipulations [203].

The hippocampal slice model enables researchers to study various aspects of epileptic activity in a controlled setting. By applying electrical stimulation or pharmacological agents to these slices, scientists can observe how epileptic seizures initiate, develop, and spread within the hippocampus [204]. This model allows for the precise examination of synaptic interactions, network properties, and changes in neuronal excitability associated with epilepsy. One of the key advantages of using hippocampal slices is the ability to directly manipulate and measure neuronal activity without the complexities of the whole brain. This approach provides detailed insights into the cellular and molecular mechanisms underlying epileptic seizures, including alterations in synaptic transmission, excitability, and the roles of various neurotransmitters and ion channels [205]. Overall, the hippocampal slice model is a valuable tool for exploring the fundamental processes involved in epilepsy and for evaluating the effects of potential therapeutic interventions [206]. It offers a focused and controlled environment for studying epileptic activity and contributes to a deeper understanding of how disruptions in hippocampal function contribute to seizure disorders. Organotypic cultures involve the cultivation of brain tissue slices in a three-dimensional environment that maintains much of the tissue’s original structure and cellular connectivity. This model provides a valuable platform for studying synaptic plasticity and neurogenesis in the context of epilepsy.

### 3.2. Brain Slices

In organotypic cultures, brain slices are prepared from various regions, such as the hippocampus, and maintained in a culture medium that supports their growth and function [207]. These cultures preserve the spatial organization and cellular interactions present in the intact brain, making them an excellent model for investigating how epileptic conditions influence neural networks. One of the key advantages of organotypic cultures is their ability to facilitate the study of synaptic plasticity, which refers to the changes in the strength and efficiency of synaptic connections in response to experience or activity [208]. By applying specific stimuli or pharmacological agents to the cultured tissue, researchers can observe how epilepsy affects synaptic transmission and plasticity. This includes examining alterations in long-term potentiation (LTP) and long-term depression (LTD), which are critical processes underlying learning, memory, and epileptic activity.

Organotypic cultures also provide insights into neurogenesis, the process by which new neurons are generated and integrated into existing neural circuits [209]. This aspect is particularly relevant for understanding how epileptic conditions impact the generation of new neurons and their functional integration. By analyzing changes in neurogenesis within these cultures, researchers can gain a deeper understanding of how epilepsy influences neuronal development and survival. Overall, organotypic cultures are a powerful tool for exploring the mechanisms underlying synaptic plasticity and neurogenesis in epilepsy [210]. They offer a more controlled and manipulable environment compared to whole-brain models, allowing for detailed examination of how epileptic conditions affect neural networks and potential therapeutic strategies aimed at modulating synaptic function and promoting neurogenesis [211].

## 4. Optogenetic Models

### 4.1. Optogenesis

This advanced technique enables the manipulation of specific neurons using light, allowing for the precise temporal and spatial induction or inhibition of epileptic seizures in animal models [212]. Optogenetics is particularly useful for studying specific neuronal circuits involved in epileptogenesis. These models have gained popularity due to their precision, control, and relevance to the human epilepsy pathophysiology, making them superior to older models like the cobalt-induced one [66]. Optogenetic epilepsy models use advanced genetic manipulation and light-based techniques to precisely control neuronal activity with high spatial and temporal resolution [67]. These models enable the induction or inhibition of seizures by selectively activating or silencing neurons, providing a powerful tool for studying the cellular mechanisms and circuits involved in epilepsy. The key mechanisms are as follows:

#### 4.1.1. Light Sensitivity via Opsins

In optogenetic models, light-sensitive proteins called opsins are used, such as channelrhodopsin (ChR2) for activation and halorhodopsin (NpHR) or archaerhodopsin (ArchT) for inhibition. These proteins are genetically inserted into specific neurons and activated by specific wavelengths of light (usually blue for ChR2 and yellow or green for NpHR and ArchT). This allows for direct modulation of the activity of epilepsy-related neurons.

#### 4.1.2. Seizure Induction by Activating Excitatory Neurons

By expressing channelrhodopsin in excitatory neurons, light pulses can depolarize these cells and cause excessive excitation in the brain. This hyperexcitation simulates the abnormal activity seen during seizures, allowing researchers to replicate epileptic episodes in specific brain regions like the hippocampus or cortex [66].

#### 4.1.3. Seizure Suppression by Controlling Inhibitory Neurons

Conversely, inhibitory opsins such as halorhodopsin or archaerhodopsin can be expressed in inhibitory neurons, like GABAergic interneurons. Activating these cells with light inhibits their activity, helping to stop ongoing seizures or prevent their initiation by restoring the balance between neuronal excitation and inhibition [66].

Optogenetics allows for precise investigation of neuronal circuits [213]. By directing opsin expression to specific types of neurons or brain areas, researchers can explore how dysfunction in certain cellular populations contributes to epilepsy. For example, activating or inhibiting neurons in the hippocampus, thalamus, or cortex enables the study of how these regions contribute to seizure generation and propagation [214]. These models also allow researchers to study synaptic plasticity in the context of epilepsy. By precisely controlling neuronal activity, it is possible to observe how chronic hyperexcitability affects synaptic strength, which could lead to the development of targeted treatments to prevent the synaptic changes associated with seizures [215]. In general, optogenetic epilepsy models are crucial for understanding the cellular and circuit mechanisms underlying epilepsy, offering a versatile approach to exploring new, more specific, and effective therapies.

## 5. Discussion

Epilepsy research relies on a variety of experimental models that have helped unravel the underlying pathophysiological mechanisms, develop new treatments, and study epileptogenesis (Table 1) [45]. Among these, genetic models have been essential in exploring the molecular and cellular bases of epilepsy [216]. Mutations in specific genes such as *GABRA1*, *GRIN2A*, and *CACNG2* have been associated with different types of epilepsy, providing valuable models for understanding how these genetic alterations affect neuronal function and trigger epileptic seizures [163]. For instance, Cossette et al. (2002) identified a mutation in *GABRA1*, which encodes a subunit of the GABA_A_ receptor, associated with juvenile myoclonic epilepsy, a type of generalized epilepsy [217]. Subsequent studies, such as Kang, Shen, and Macdonald (2015), explored how this mutation affects GABA_A_ receptor current and stability, offering detailed insight into how alterations in inhibitory neurotransmission predispose individuals to seizures. Similarly, mutations in *GRIN2A*, which encodes a subunit of the NMDA receptor, have been linked to epileptic encephalopathies, as described by Endele et al. (2010) [218]. This model has been crucial for studying how NMDA receptor dysfunction contributes to neuronal hyperexcitability, resulting in severe seizures and developmental disorders. 

The Stargazer model, with mutations in *CACNG2* [219], has been instrumental in understanding absence epilepsy. Letts et al. (1998) demonstrated that this mutation affects voltage-gated calcium channels in the brain, altering glutamatergic neurotransmission and contributing to the genesis of absence seizures [219]. However, a significant limitation of these genetic models is that they often reflect only a part of the complex network of genetic and environmental interactions that cause epilepsy in humans. Additionally, genetic variability between species presents challenges for direct clinical translation. In parallel, chemical models are widely used due to their ability to reliably and reproducibly induce seizures and status epilepticus. Among these, the pilocarpine, kainate, KA, and PTZ models have been pivotal in studying epileptogenesis, studying the structural and functional changes in the brain, and evaluating new anticonvulsant treatments [1]. The pilocarpine model [220], for instance, is one of the most commonly used to study temporal lobe epilepsy. Turski et al. (1983) demonstrated that pilocarpine administration induces status epilepticus [221], followed by a chronic phase characterized by recurrent spontaneous seizures. This model has been instrumental in understanding how neuronal damage in the hippocampus and other brain regions contributes to chronic epilepsy. Similarly, kainate and KA are convulsants used to induce temporal lobe epilepsy, as shown by Ben-Ari (1985) and Cavalheiro et al. (1991) [222,223]. They demonstrated that kainate administration causes seizures and specific neuronal damage in the hippocampus, making it an ideal model for studying the underlying mechanisms of neuronal damage associated with epilepsy [224]. These models have been fundamental in understanding excitotoxicity and neurodegeneration in the context of epilepsy. On the other hand, PTZ, a GABA_A_ receptor antagonist, is primarily used to study generalized seizures. Löscher and Hönack (1993) showed that PTZ administration induces clonic and tonic seizures, making it useful for evaluating the efficacy of new anticonvulsants [225,226]. However, a limitation of this model is that it does not fully reproduce the complexity of human epilepsy, as it focuses on a single mechanism of action.

**Figure 1:**
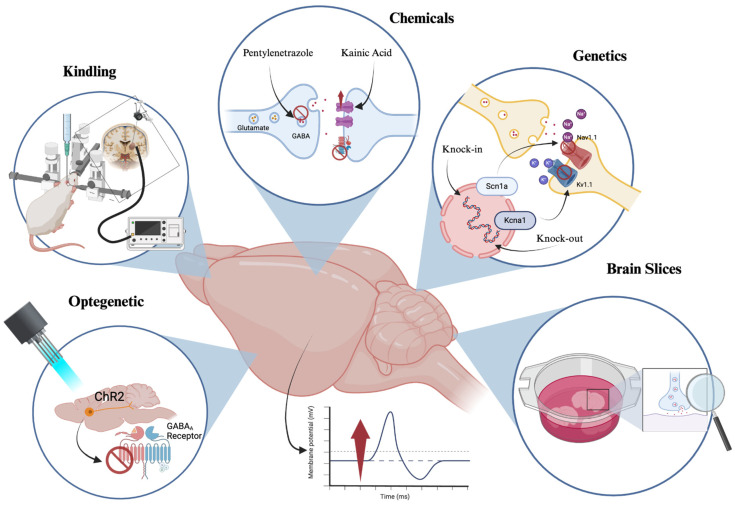
The most representative epilepsy models currently in use. Created with https://www.biorender.com (accessed on 20 August 2024).

The new epilepsy models based on PTZ, along with other chemical and genetic models in zebrafish, promise significant advances and the opening of new research opportunities due to the multiple benefits they offer. However, it should not be forgotten that, although the phylogenetic homology is quite similar, it does not perfectly match that of humans [174,175,176,177]. Additionally, the anatomical structures of zebrafish larvae are incomplete and cannot be fully compared to those of humans [137,138]. In summary, these models have been fundamental in advancing the understanding of epilepsy (Figure 1), allowing the elucidation of the neuroinflammatory and molecular effects underlying a multitude of proteins and genes related to various cellular pathways involved in it. Although each of these models has its limitations, the need for a multifaceted approach that integrates different models is emphasized to obtain a more comprehensive view of this complex pathology. Though each has its limitations. This highlights the need for a multifaceted approach that integrates different models to gain a more comprehensive view of this complex pathology.

## 6. Conclusions

The epilepsy models discussed have been fundamental in advancing our understanding of this complex disorder. Genetic models provide a window into the molecular foundations of epilepsy, while chemical models offer well-controlled systems for studying epileptogenesis and testing potential therapies. Prenatal stress models expand our understanding of how environmental factors may influence the risk of developing epilepsy. However, each model has its limitations. Genetic models, while revealing, often do not capture the full complexity of human epilepsy. Chemical models, though useful for inducing seizures, may oversimplify the disease mechanisms. Prenatal stress models, while offering a unique perspective, are difficult to standardize and replicate. Thus, future research should focus on integrating data from multiple models, combining genetic, chemical, and environmental approaches to develop a more comprehensive and holistic understanding of epilepsy. This integrated approach will not only deepen our understanding of the underlying mechanisms but also enable the development of more effective and personalized treatments for epilepsy patients.

## Figures and Tables

**Table 1:** Summary of experimental models used in the study of epilepsy, organized by type (genetic, chemical, physical, or in vitro). The table details the specific mechanism or mutation of each model, along with its primary research application, providing a clear understanding of how each model contributes to the study of different forms of epilepsy and their respective pathological mechanisms.

Model	Mechanism/Mutation	Main Use/Type of Epilepsy
In Vivo Models		
Genetic Models		
*Papio papio*	Cortical hyperexcitability induced by visual stimuli	Photosensitive Seizures
Gerbils	Auditory stimuli	
Scn1a KO or Scn1a+/−	Sodium channel Nav1.1	Dravet Syndrome
Scn2a	Sodium channel Nav1.2	Infantile Generalized Epilepsy
Scn8a (D/+ or N1768D Mice)	Sodium channel Nav1.6	Focal and Generalized Epilepsy
Kcna1 KO	Potassium channel Kv1.1	Benign Partial Epilepsy with Febrile Seizures Plus
Cacna1a (Tottering Mice)	Calcium channel Cav2.1	Absence Epilepsy, Ataxia
Stargazer mice	Cacng2 Gene	Absence Epilepsy
The Ducky (Ddu) mice	Cacna2d2 Gene	
Gabra1	GABA_A_ Receptor	Juvenile Myoclonic Epilepsy
Grin2a	NMDA Receptor	Focal Epilepsy and Epileptic Encephalopathy
PTEN	PTEN Gene	Epilepsy Associated with Tuberous Sclerosis
Ch2	Ch2d2 Gene	Lennox–Gastaut Syndrome, Myoclonic–Atonic Epilepsy, and Dravet Syndrome
Chemical Models		
Kainic Acid	Kainate Derivative	Temporal Lobe Epilepsy and Selective Neuronal Death
Pentylenetetrazol (PTZ)	GABA_A_ Receptor Antagonist	Generalized Seizures
Electrical Models		
Kindling	Repeated electrical stimulation	Temporal Lobe Epilepsy
Structural Models		
Hippocampal Sclerosis	Hippocampal injury	Temporal Lobe Epilepsy
In Vitro Models		
Hippocampal Slices	Isolated brain tissue	Study of Epileptic Activity
Organotypic Cultures	Brain tissue cultures	Synaptic Plasticity and Neurogenesis in Epilepsy
Optogenetic	Light-sensitive proteins (opsins); ChR2 for activation and NpHR or ArchT for inhibition	Seizure Induction by Activating Excitatory Neurons and Seizure Suppression by Controlling Inhibitory Neurons

## Data Availability

All clinical and statistical data and materials are available for the benefit of science.
