# Correction: Rubio et al. Classification of Current Experimental Models of Epilepsy. *Brain Sci.* 2024, *14*, 1024

**DOI:** 10.3390/brainsci16040400

**Published:** 2026-04-09

**Authors:** Carmen Rubio, Héctor Romo-Parra, Alejandro López-Landa, Moisés Rubio-Osornio

**Affiliations:** 1 Department of Neurophysiology, Instituto Nacional de Neurología y Neurocirugía, Mexico City 14269, Mexico; macaru4@yahoo.com.mx (C.R.); hector.romo.parra@gmail.com (H.R.-P.); alexlanda10@hotmail.com (A.L.-L.); 2 Psychology Department, Universidad Iberoamericana, Mexico City 01219, Mexico; 3 Department of Neurochemistry, Instituto Nacional de Neurología y Neurocirugía, Av. Insurgentes Sur 3877, Mexico City 14269, Mexico

**Text Correction**

There was an error in the original publication [1]. The DBA/2J and C57BL/6 strains were incorrectly attributed to gerbils. These are mouse strains and are not discussed in the cited reference.

A correction has been made to Section 2.1.1:

“Nevertheless, individual strains of gerbils exhibit varying degrees of vulnerability to audiogenic seizures, which has supported their use in epilepsy research [71].”

The authors state that the scientific conclusions are unaffected. This correction was approved by the Academic Editor. The original publication has also been updated.

## References

[1] Rubio C., Romo-Parra H., López-Landa A., Rubio-Osornio M. (2024). Classification of Current Experimental Models of Epilepsy. Brain Sci..

